# Single spike mutation differentiating XBB.1 and XBB.1.5 enhances SARS-CoV-2 cell-to-cell transmission and facilitates serum-mediated enhancement

**DOI:** 10.3389/fimmu.2024.1501200

**Published:** 2024-11-27

**Authors:** Elena Criscuolo, Benedetta Giuliani, Matteo Castelli, Mattia Cavallaro, Sofia Sisti, Roberto Burioni, Davide Ferrari, Nicasio Mancini, Massimo Locatelli, Nicola Clementi

**Affiliations:** ^1^ Laboratory of Microbiology and Virology, Vita-Salute San Raffaele University, Milan, Italy; ^2^ SCVSA Department, University of Parma, Parma, Italy; ^3^ Laboratory of Medical Microbiology and Virology, Fondazione Macchi University Hospital, Varese, Italy; ^4^ IRCCS San Raffaele Scientific Institute, Milan, Italy

**Keywords:** SARS-CoV-2, Spike, COVID-19, XBB.1, XBB.1.5, neutralizing antibodies, fusion

## Abstract

**Introduction:**

The ongoing emergence of SARS-CoV-2 variants poses significant challenges to existing therapeutics. The spike (S) glycoprotein is central to both viral entry and cell-to-cell transmission via syncytia formation, a process that confers resistance to neutralizing antibodies. The mechanisms underlying this resistance, particularly in relation to spike-mediated fusion, remain poorly understood.

**Methods:**

We analyzed two clinical SARS-CoV-2 isolates differing by a single amino acid substitution in the S protein. Using biochemical and cell-based assays, we evaluated entry kinetics, syncytia formation, and the neutralizing efficacy of convalescent sera. These parameters were further correlated with S-mediated cell-cell fusion activity.

**Results:**

The single amino acid substitution significantly altered entry kinetics and enhanced syncytia formation. This modification did not diminished the neutralizing capacity of convalescent sera, but it increased the efficiency of S-induced cell-cell fusion. These findings highlight the mutation’s impact on viral transmissibility and immune evasion.

**Discussion:**

Our study demonstrates that even minor changes in the S protein can profoundly influence SARS-CoV-2 transmissibility and resistance to antibody-mediated neutralization. Understanding the molecular basis of S-mediated cell-cell fusion is crucial for anticipating the impact of emerging variants and developing next-generation therapeutic strategies. These insights provide a framework for predicting variant fitness and optimizing treatment approaches against future SARS-CoV-2 variants.

## 1 Introduction

Since the onset of the COVID-19 pandemic, the emergence of new variants of SARS-CoV-2 has garnered global attention, prompting researchers and healthcare professionals to closely monitor their characteristics and implications for public health. In November 2021, the BA.1 variant (progenitor of Omicron lineage) appeared in Africa and swiftly became the predominant circulating variant worldwide (1, 2). The spike protein alone harbored at least 30 amino acid substitutions (including 15 mutations in the receptor-binding domain, RBD), accounting for its heightened transmissibility and reduced vaccine efficacy compared to other variants of concern (VOCs) (3). Over time, a complex landscape of Omicron sublineages and variants has evolved through mutation and recombination, each bearing a distinct genetic signature (3, 4). The substantial mutation load characteristic of Omicron variants may also result in altered processing of the spike protein by cellular enzymes, conceivably affecting their ability to engage in cell-to-cell spreading and potentially implicating an additional immune evasion mechanism (5, 6). Amongst them, XBB.1 and XBB.1.5 globally circulated in 2023, with the latter being predominant. They exhibited increased transmissibility and immune evasion capabilities compared to previous Omicron variants (7–9).

XBB.1 and XBB.1.5 are extremely similar throughout the genome and differ just for a proline instead of a serine at position 486 in the sequence of spike (S) protein (10). *In silico* analysis has revealed that this additional mutation may enhance the binding affinity of the XBB.1.5 RBD to human ACE2 compared to XBB.1, potentially explaining how it managed to replace the previous variant at an epidemiological level (11). The significantly higher binding affinity of the XBB.1.5 RBD to ACE2 was also confirmed through biolayer interferometry experiments and yest surface display assays (12, 13). Furthermore, similar results were observed using surface plasmon resonance, highlighting the impact of the S486P substitution on the binding affinity of RBD to the host receptor (8, 13).

The distinct antigenic signature of these two Omicron variants has raised concerns regarding their potential negative impact on the efficacy of the prevention and therapeutical measures against COVID-19 pandemic. Data published over one year of the widespread use of the FDA approved vaccines and monoclonal antibodies (mAbs) confirmed a decline in their efficacy related to the spread of new SARS-CoV-2 VOCs (14, 15). XBB.1 and XBB.1.5 make no exception (7, 8, 16), as plasma from subjects who received three doses of Wuhan-based mRNA vaccine failed to neutralize both XBB.1 and XBB.1.5 in *in vitro* infection studies, displaying a similar immune-evasive feature (8, 17, 18). The dramatic frequency of breakthrough infections prompted the release of updated COVID-19 vaccines encoding the S protein of XBB.1.5, with efficacy validated by a notable enhancement in neutralizing capacity against Omicron variants compared to sera from individuals vaccinated with the earlier formulations (19, 20).

The complex landscape of mutations in Omicron variants may also compromise the efficacy of therapeutic mAbs, which can vary dramatically against specific variants and subvariants (8, 17, 21, 22). Reports revealed either the lack of neutralizing capability or a reduction in the effect of the main therapeutical mAbs (8, 17). These data suggest that the S486P mutation does not affect the immune evasion capability of XBB.1.5, which retains the same characteristics as XBB.1 (8, 12). Considering the remarkable reduction of activity against the novel Omicron variants, the COVID-19 Treatment Guidelines recommends against the use of the current mAbs to treat SARS-CoV-2 infection in clinical practice (23).

This work will discuss the *in vitro* key differences between XBB.1 and XBB.1.5 clinical isolates in terms of entry efficiency, fusogenic capability, and susceptibility to sera from recently recovered individuals, assayed for their entry and post-entry neutralization activity. Our results highlight how a single spike mutation can significantly alter SARS-CoV-2 fitness through an improved cell-to-cell transmissibility that exploits antibody-mediated spike enhancement.

## 2 Materials and methods

### 2.1 Clinical samples

Twenty-nine serum samples from healthcare professionals from San Raffaele Hospital were collected as part of the CE:199/INT/2020 study approved by the San Raffaele Hospital, Milan, Italy, Institutional Ethical Review Boards. Written informed consent was obtained from all participants.

### 2.2 Cell lines

Vero E6 (Vero C1008, clone E6; ATCC CRL-1586) cells and 293T-ACE2.TMPRSS2 (*Homo sapiens* Embryonic Kidney Epithelial Cells Expressing Transmembrane Protease Serine 2 and Human Angiotensin-Converting Enzyme 2, BEI Resources, NIAID, NIH: NR-55293) were cultured in Dulbecco’s modified Eagle’s medium (DMEM) supplemented with non-essential amino acids (NEAA), penicillin/streptomycin (P/S, 100 U/mL), HEPES buffer (10 mM) and 10% (v/v) heat- inactivated fetal bovine serum (FBS). We added 1 mg/mL of Geneticin (G418) to the medium of Vero E6 that stably expressed TMPRSS2 (Vero E6-TMPRSS2, NIBSC 100978). Calu-3 (Human lung cancer cell line, ATCC HTB-55) cells and Caco-2 cells (Human epithelial colorectal adenocarcinoma cells, ATCC HTB-37) were cultivated in Minimum Essential Medium (MEM) supplemented with NEAA (1×), P/S (100 U/mL), 1 mM sodium pyruvate and 10% (v/v) heat-inactivated FBS. All cell lines were incubated at 37°C and 5% CO_2_ in a humidified atmosphere. All cell lines were routinely tested for mycoplasma (Lonza, LT07-218).

### 2.3 Viruses

Two clinical isolates of SARS-CoV-2 were obtained and propagated in Vero E6-TMPRSS2 cells: XBB.1 (GISAID accession ID: EPI_ISL_ 16526278) and XBB.1.5 (GISAID accession ID: EPI_ISL_18537906).

In detail, 0.8 mL of the transport medium of the nasopharyngeal swab (COPAN’s kit UTM^®^ universal viral transport medium—COPAN) was mixed 1:1 with DMEM without FBS and supplemented with P/S and Amphotericin B. The mixture was added to an 80% confluent Vero E6 cell monolayer seeded in a 25 cm^2^ tissue culture flask. After 1 h adsorption at 37°C, 3 mL of DMEM supplemented with 2% FBS and Amphotericin B was added. One day post-infection (dpi), the monolayer was washed in PBS 1×, and 4 mL of DMEM supplemented with 2% FBS and Amphotericin B was added. The cytopathic effect was monitored using inverted phase-contrast microscopy (Olympus CKX41), and the supernatant was collected at monolayer complete disruption.

The viral RNA was extracted from 200 µL of heat inactivated viral stock using the ELITe InGenius™ workstation (ELITechGroup). Targeted RNA enrichment for Illumina sequencing was then performed using the BS-Paragon CleanPlex SARS-CoV-2 Flex Panel (Paragon Genomics) following the manufacturer’s instructions. The purified genetic material was quantified using the Qubit double-stranded DNA (dsDNA) HS Assay Kit on a Qubit 2.0 Fluorometer (Thermo Fisher) and samples were diluted and pooled to a final concentration of 2 nM. Then, 50 pM of the pooled library was sequenced on the iSeq Illumina platform using the iSeq 100 i1 Reagent v2 (300-cycle).

The obtained data were then analyzed using the coronavirus antiviral and resistance database of Stanford University (https://covdb.stanford.edu/sierra/sars2/by-patterns/) through which the consensus sequence of XBB.1 and XBB.1.5 viral stock was obtained. Then the fasta sequences were also verified through Nextclade software (https://clades.nextstrain.org).

### 2.4 Virus titration

Virus stocks were titrated using an Endpoint Dilutions Assay (EDA, TCID_50_/mL). Vero E6 cells were seeded into 96-well plates and infected at 95% of confluency with base 10 dilutions of virus stock. After 1 h of adsorption at 37°C, the cell-free virus was removed, cells were washed with PBS 1×, and a complete medium was added to cells. After 72 h, cells were observed to evaluate the presence of a cytopathic effect (CPE). TCID_50_/mL of viral stocks were then determined with the Reed–Muench formula.

### 2.5 Kinetic profiles

Vero E6, Vero E6-TMPRSS2, Calu-3 and Caco-2 (3 × 10^5^ cells/mL) cells were seeded in 96-well plates and cultured for 1 day at 37°C and 5% CO_2_ in a humidified atmosphere. Then, the cells were infected with the two different SARS-CoV-2 variants (0.001 multiplicity of infection, MOI) in triplicate. After 1h of adsorption, cells were washed three times with PBS 1× to remove cell-free virus, and fresh medium was added. Cell supernatants were collected at 4 time points: 6, 24, 48 and 72 hours post infection (hpi), and viral RNA was purified from cell culture supernatant using the QIAamp Viral RNA Mini Kit (QIAGEN, Hilden, Germany), following the manufacturer’s instructions. Subsequently, the purified RNA was used as template to synthesize the first-strand cDNA, using the SuperScript™ First-Strand Synthesis System for RT-PCR (Thermo Fisher Scientific, 11904018), following the manufacturer’s instruction. Real-time PCR, using SYBR^®^ Green dye-based PCR amplification and detection method, was performed to detect the cDNA. We used the SYBR™ Green PCR Master Mix (Thermo Fisher Scientific, 4309155), the forward primer N2F: TTA CAA ACA TTG GCC GCA AA, the reverse primer N2R: GCG CGA CAT TCC GAA and the following PCR conditions: 95°C for 2 min, 45 cycles of 95°C for 20 s, annealing at 55°C for 20 s and elongation at 72°C for 30 s, followed by a final elongation step at 72°C for 10 min.

Real Time-PCR results were analyzed calculating Delta (Δ) Ct as the difference between Ct values obtained for the different time points and Ct (6 h).

### 2.6 Pseudovirus entry assay

We generated lentiviral pseudoviruses following the protocol already described (24). Briefly, 75 cm^2^ tissue culture flask of 60% confluent HEK-293T cells was transfected using PEI Prime™ linear (Sigma-Aldrich, 919012-100MG) with five plasmids: pHAGE with CMV-driven Luciferase-IRES-ZsGreen (BEI Resources, NIAID, NIH: NR-52516), pHDM with HIV Gag-Pol (BEI Resources, NIAID, NIH: NR-52517), pHDM with HIV Tat (BEI Resources, NIAID, NIH: NR-52518), pRC with CMV-driven HIV Rev (BEI Resources, NIAID, NIH: NR-52519) and either mammalian expression vector pcDNA3.3 harboring the S gene of either XBB.1.5 (Addgene, #196585) or XBB.1 (obtained from site-direct mutagenesis from the previous plasmid using the primers P482S_fwd: 5’-TGGCGGGCAGCAACTGTTAC; P482S_rev 5’-CATTACATGGTTTGTTGCCAGCC). At 72 h post-transfection, cell supernatant was harvested, centrifuged at 4500 rpm for 20 minutes to eliminate cell debris. Pseudoparticles in the media were subsequently pelleted by ultracentrifugation through a 20% sucrose cushion at 20,000 rpm for 1 h by using Beckman 328 SW28 rotor. Pseudoviruses were aliquoted and stored at −80°C before titration. In detail, pseudovirus dilutions were added to the Vero E6/TMPRSS2 cells, and spinoculation (1 h at 800 g) was performed to allow pseudovirus adsorption. Subsequently, media was removed, cells were washed with PBS 1×, and a complete medium was added to cells. 72 h post-infection, the cells were lysed with 100 μL of Glo Lysis Buffer (Promega, Madison, WI, USA) for 15 min at room temperature. Cell lysates were transferred to a luminometer plate and 100 μL of Bright-Glo Assay Reagent (Promega) was added immediately before detection (Victor3, Perkin Elmer). We performed six biological replicates for each condition.

For pseudovirus entry assays, Calu-3 cells were seeded in 96-well plates and cultured at 37°C and 5% CO_2_ in a humidified atmosphere. Equal doses of XBB.1 and XBB.1.5 pseudovirus particles were added to cell monolayers, and after spinoculation the cells were incubated for 72 hours at 37°C and 5% CO_2_. Luciferase activity was detected as described above.

### 2.7 Virus attachment and entry assay

Vero E6 and Vero E6-TMPRSS2 (10^5^ cells/mL) cells were seeded on Matrigel^®^ coated slides with a removable 12-well silicone chamber (Ibidi). The following day, the cells were chilled with cold PBS 1× and then infected with 1 MOI of either XBB.1 or XBB.1.5 for 30 minutes on ice. For the attachment assay, the viral inoculum was removed, and after a wash with cold PBS 1×, the cells were immediately fixed with 4% PFA for 15 min at room temperature. For the viral entry assay, after the 30 minutes on ice, the slides were incubated for 30 minutes at 37°C and fixed as previously described. To distinguish extracellular and intracellular viral particles, we used a two-step dual staining procedure as described elsewhere (25). The fixed cells were blocked using 5% BSA in PBS 1× for one hour, followed by staining with anti-S protein (Sino Biological, 40592-MM117) for 30 minutes at 37°C, washed and stained with goat anti-mouse IgG Alexa Fluor 488 (Invitrogen, A-11001) for 30 minutes at 37°C, then washed and fixed with 4% PFA for 15 minutes at RT. The cells were blocked and permeabilized using 5% BSA + 0.3% triton X100 in PBS 1× for 1 h at 37°C, stained with anti-N protein (Sino Biological, 40143-MM05) for 1 h at 37°C, then washed and stained with goat anti-mouse IgG Alexa Fluor 546 (Invitrogen, A-11003) and Phalloidin 647 (Invitrogen, MAN0001777) for 30 minutes at 37°C. Nuclei were stained using Hoechst 33258 (Sigma-Aldrich, 94403) for 15 minutes at 37°C. Five fields for each image were acquire using Olympus FluoVIEW FV3000RS Confocal microscope and ImageJ software (Rasband, ImageJ, U.S.N.I.H., Bethesda USA, http://imagej.nih.gov/ij/) was used to analyze the red area. Virus adsorption events were counted as objects with a diameter range of 1-15 pixels using CellProfiler (www.cellprofiler.org) (26).

### 2.8 Plaque assay

Vero E6 and Vero E6-TMPRSS2 cells were seeded in 24-well TC-treated plates and cultured for 1 day at 37°C and 5% CO_2_ in a humidified atmosphere. Confluent monolayers of cells were infected with five 10-fold dilutions of either XBB.1 or XBB.1.5. After 1 h of adsorption at 37°C, the cell-free virus was removed, and the cells were washed with PBS 1×. Cells were then incubated for 46 h in DMEM containing 2% FBS and 1% Agarose. Cells were fixed and stained with crystal violet dye, and images were acquired using Zeiss Axio Observer.Z1 microscope with QImaging Exi-Blue. Viral plaques were counted, and their area was measured using ImageJ software.

### 2.9 Immunofluorescence of virus-induced syncytia formation

Calu-3 cells were seeded in 12-well silicone chamber (Ibidi), and the experiment was performed at 70% confluency. Cells were infected with 0.1 MOI of XBB.1 or XBB.1.5 for 1 h at 37°C, then washed with PBS 1×, and incubated in complete medium supplemented with 2% at 37°C in the presence of CO_2_ for 72 h. Then were washed with PBS 1× and fixed with MeOH: Ac (1:1) for 15 minutes. After rinsing the cells with two PBS 1× washes, the cells were stained with a-ZO-1 antibody (Invitrogen, 33-9100). After a PBS 1x wash, the cells were stained with goat anti-mouse IgG (H+L) cross-adsorbed secondary antibody, Alexa Fluor 488 (Thermo Fisher Scientific, A-11001). Hoechst 33258 (Sigma-Aldrich, 94403) was used for nuclear staining, and images were acquired using Olympus FluoVIEW FV3000RS Confocal microscope.

### 2.10 Cell fusion reporter system

To study the cell-cell fusion activity, we used a reporter system developed elsewhere (27). The mNeonGreen split protein and NanoLuc split protein with a pair of interacting leucine zippers (bFos-bJun) were cloned in into eukaryotic expression plasmid pcDNA3.1 using XbaI and XhoI restriction enzymes. In detail, the mNeonGreen protein was split into NG (1–173 amino acids) and CG (174–236 amino acids), and NanoLuc was split into LgBiT (1–159 amino acids) and SmBiT (160–171 amino acids). NG and SmBit were fused to different terminals of bJun named NGJS or SJNG, whereas CG and LgBit were fused to different terminals of bFos named CGFL or LFCG. To evaluate the different combinations of the reporter system, we transfected 293T-ACE2.TMPRSS2 cells seeded in a 6-well plate with the four different plasmids (NGJS, SJNG, CGFL, LFCG) individually or co-transfected with SJNG and CGFL, SJNG and LFCG, NGJS and LFCG, and NGJS and CGFL. The next day the cells in each well were detached and resuspended in fresh DMEM containing 10% FBS. 4 × 10^4^ target cells were reseeded in a 96-well plate and incubated for 24 h at 37°C. 48 h after seeding, the cells luciferase activity was evaluated using the Nano-Glo Luciferase Assay Kit as manufacturer’s instruction (Victor3, Perkin Elmer).

### 2.11 Cell fusion assay

The cell-cell fusion activity was analyzed using the previously described reporter system. Briefly, 293T-ACE2.TMPRSS2 cells were seeded in a 6-well plate, and 24 h later, the cells were transfected with either NGJS or CGFL using Lipofectamine 2000 transfection reagent (Invitrogen, 11668019). After incubation at 37°C for 24 h, the cells in each well were detached and resuspended in fresh DMEM containing 10% FBS. 4 × 10^4^ target cells were reseeded in a 96-well plate and incubated for 24 h at 37°C. For the S-based syncytia formation, the co-colture was transfected with pcDNA3.3 harboring either the S gene of either XBB.1.5 (Addgene, #196585) or XBB.1 (obtained from site-direct mutagenesis as previously described) using Lipofectamine 2000 transfection reagent (Invitrogen, 11668019). Alternatively, for the cell fusion assay with the clinical isolates, the co-culture was infected with different MOI of XBB.1 or XBB.1.5 for 1 h at 37°C. 24 hours later, the mNeonGreen protein was detected using fluorescent microscopy (Zeiss Axio Observer.Z1 microscope with QImaging Exi-Blue), and integrated density (area × mean gray value) was used to compare fluorescence intensities. Luciferase activity was detected using the Nano-Glo Luciferase Assay Kit as manufacturer’s instructions (Victor3, Perkin Elmer).

### 2.12 Microneutralization assay

Vero E6 were seeded into 96-well plates 24 h before the experiment performed at 95% cell confluency for each well. Serum samples were decomplemented by incubation at 56°C for 30 min, and serial dilutions were incubated with SARS-CoV-2 variants at 0.01 MOI for 1 h at 37°C. Virus–serum mixtures and positive infection control were applied to cells monolayers after a PBS 1× wash, and virus adsorption was performed at 37°C for 1 h. Then, the cells were washed with PBS 1× to remove cell-free virus particles and virus-containing mixtures and controls were replaced with complete medium supplemented with 2% FBS. The plates were incubated at 37°C in the presence of CO_2_ for 72 h. The experiments were performed in triplicate. Neutralization activity was evaluated by comparing CPE presence detected in the presence of virus–serum mixtures to positive infection control. The scoring system used was as follows: 0 = uninfected; 0.5 to 2.5 = increasing numbers or areas of plaques; 3 = all cells infected. A score of 3 (full infection) represented 0% infection inhibition, while a score of 0 (no infection) indicated 100% infection inhibition. The whole surface of the wells was considered for the analysis (5× magnification) in inverted phase-contrast microscopy (Olympus CKX41).

### 2.13 Post entry inhibition assay

The post entry inhibition assay (PEI) was performed using the reporter system previously described (28). 293T-ACE2.TMPRSS2 cells were seeded in a 6-well plate, and 24h later the cells were transfected using Lipofectamine 2000 transfection reagent (Invitrogen, 11668019) with either NGJS or CGFL. After incubation at 37°C for 24 h, the cells in each well were detached and resuspended in fresh medium containing 10% FBS. 4 × 10^4^ target cells were reseeded onto a 96-well plate and incubated for 24 h at 37°C. The co-culture was infected with 0.01 MOI of XBB.1 or XBB.1.5 for 1 h at 37°C. After viral adsorption, cell-free virus was removed, and cells were incubated for 24 h in DMEM containing 2% FBS and two dilutions of decomplemented serum (1:20 or 1:40). The luciferase activity was detected using the Nano-Glo Luciferase Assay Kit as manufacturer’s instructions (Victor3, Perkin Elmer).

### 2.14 Real-Time qPCR analysis of ACE2 and TMPRSS2 expression levels

Cellular RNA from Vero E6, Vero E6-TMPRSS2, Calu-3, Caco-2, 293T-ACE2.TMPRSS2 (2 × 10^6^ cells) were extracted using the RNeasy Mini Kit (QIAGEN) according to the manufacturer’s protocol. Then, the mRNA from each sample was reverse transcribed using the SuperScript™ III First-Strand Synthesis System for RT-PCR (Thermo Fisher Scientific, 18080051), following the manufacture’s instruction. We analyzed 10 ng of cDNA to evaluate the expression levels of ACE2 and TMPRSS2 with Real Time RT PCR using the SYBR^®^ Green dye-based PCR amplification and detection method. Gene-specific primers for human ACE2 (FW: AAA CAT ACT GTG ACC CCG CAT; RE: CCA AGC CTC AGC ATA TTG AAC A), monkey ACE2 (FW: AAA CAT ACT GTG ACC CCG CAT; RE: GCT TCA GCA TAT TGA GCA ATT TCT G) and human TMPRSS2 (FW: AAT CGG TGT GTT CGC CTC TAC; RE: CGT AGT TCT CGT TCC AGT CGT) were used. As endogenous control, we used β-actin (FW: CCC TGG ACT TCG AGC AAG AG; RE: ACT CCA TGC CCA GGA AGG AA). Amplification was performed under the following conditions: 94°C for 5 min, 45 cycles of 94°C for 30 s, annealing at 60°C for 30 s and elongation at 68°C for 30 s, followed by a final elongation step at 72°C for 10 min. Samples were run in triplicate in a total volume of 20 µL using ABI-PRISM 7900HT Fast Real-Time instruments (Applied Biosystems).

### 2.15 Molecular dynamics simulations

The spike systems were constructed and simulated as described in (26). Briefly, XBB.1 and XBB.1.5 ectodomains (residues 14-1142) were modeled in the closed and 1up states both as S0 and S1-S2 (processed at the furin cleaveage site – FCS) using the spike sequence from the clinical isolates described in this study as query and the deposited structures with RCSB ID: GZGI and 7BNN as templates. The last 5 ns of each 200 ns trajectory were used for stability and accessible surface area (ASA) calculations. The propensity to open the RBDs was calculated as the stability ratio between the open and closed protomers. Spike processing by furin and TMPRSS2 was estimated measuring FCS ASA (residues 671-685) in S0 and S2’ ASA (residues 801-815) in S1-S2 using a probe with radius 18.6 Å in (27).

### 2.16 S1 shedding evaluation

HEK-293T cells were seeded in a 96-well plate, and 24 h later, the cells were transfected with mammalian expression vector pcDNA3.3 harboring the S gene of either XBB.1.5 (Addgene, #196585) or XBB.1 (obtained from site-direct mutagenesis as previously described). 72 hours post-transfection, cells were incubated with 1:20 sera dilution or 12.5 nM of anti-S monoclonal antibody. Supernatants containing shedded S1 were collected 2h after incubation, added to 4× LDS sample buffer (Thermo Fisher Scientific, B0008) and boiled for 5 minutes at 95°C, then electrophoresed by SDS-Page at 200 V for 45 min in MES 1× buffer (Thermo Fisher Scientific, B0002). Proteins were transferred to a polyvinylidene difluoride (PVDF) membrane at 4°C for 2h in ice-cold Western Transfer Buffer (25 mM Tris, 192 mM Glycine, MeOH 20% (v/v)). Membrane was blocked in PBS supplemented with 5% non-fat dry milk in PBS containing 0.1% Tween-20 (PBS-T) before the incubation with primary anti-S (Sino Biological, 40150-R007) for 1h. The membranes were washed 3 times in PBS-T, followed by probing with horseradish peroxidase (HRP)-conjugated anti-rabbit antibody (Thermo Fisher Scientific, 31460) as secondary. Signal was developed by treating membranes with SuperSignal West Pico Plus Chemiluminescent Substrate (Thermo Fisher Scientific, 34580) imaging on a ChemiDoc MP System (Bio-Rad #12003154, Hercules, CA, USA).

### 2.17 Statistical analyses

Data analysis was performed using GraphPad Prism 10 (GraphPad Software, San Diego California USA, www.graphpad.com). For kinetic experiments, Real Time-PCR results were analyzed calculating Delta (Δ) Ct as the difference between Ct values obtained for experimental settings and infection control. Two-way ANOVA and Tukey’s multiple comparisons were performed to analyze the results. ΔCt was calculated as the difference between Ct values obtained for the different time points and Ct_6hpi_. Two-way Anova with Sidak’s multiple comparison test was also used to analyze the number of plaques of infected cells, the values of RLU from infected cells and the cellular receptors gene expression profile. The same statistical test was used to analyze the validation of the cell-cell reporting system. Mann-Whitney *t* test was used for statistical analysis of data obtained from pseudovirus assay, viral attachment and entry assays, the area of plaques of infected cells, the quantification of the fusogenic events by green fluorescent signal, the quantification of viral particles in viral stocks by Real-Time PCR, and the comparison between NT and PEI results obtained from 1:20 sera dilution against the two variants. Gaussian distribution was fitted to frequency distribution of the area of plaques of infected cells. The relationship between numerical variables of neutralization, post-entry inhibition and S1 shedding results was evaluated by means of Pearson and Spearman correlation analyses.

## 3 Results

### 3.1 A single S mutation enhances XBB.1.5 infection by impacting the initial molecular mechanisms involved in viral entry

**Figure 1:**
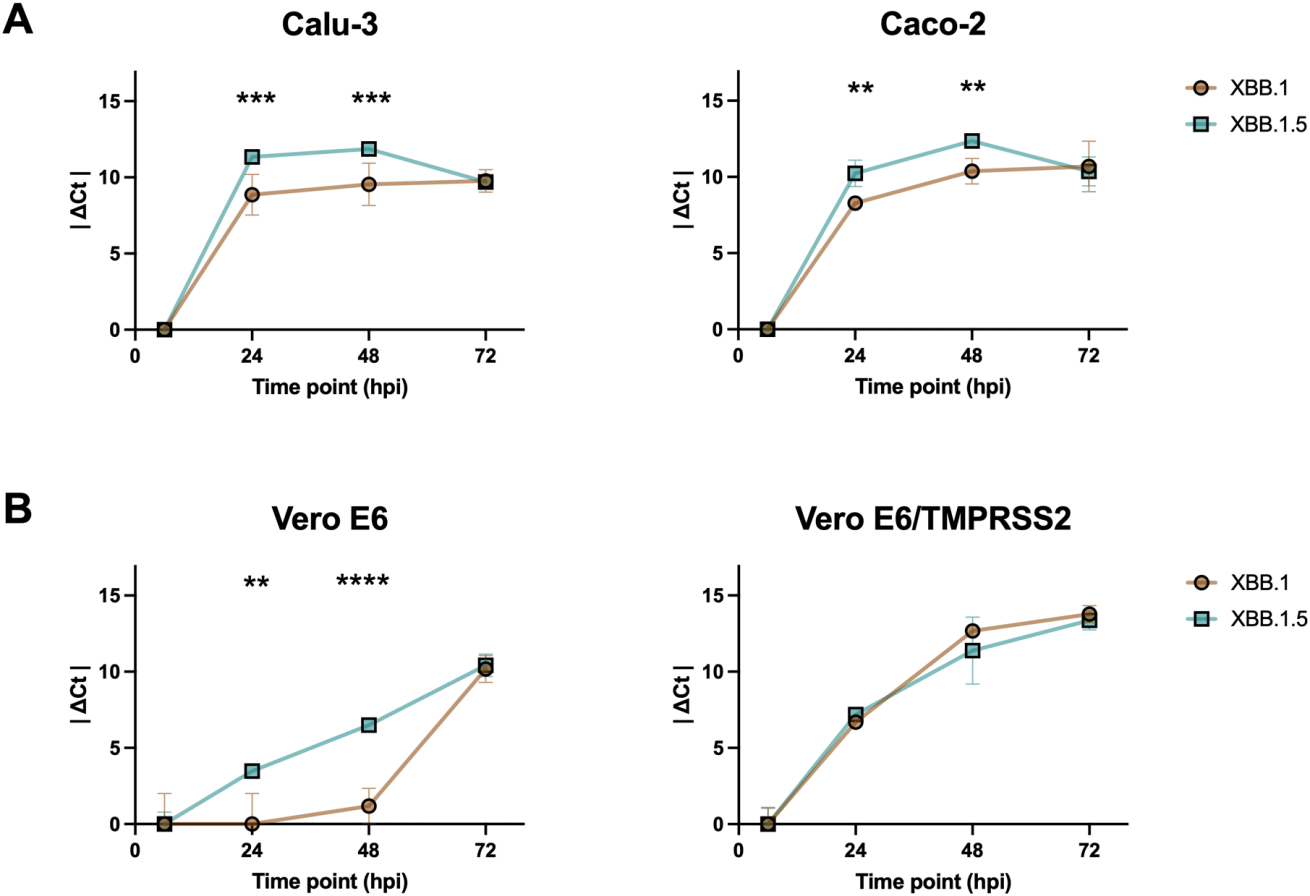
Differences between XBB.1 and XBB.1.5 infection kinetics. SARS-CoV-2 replication kinetics in **(A)** Calu-3 and Caco-2 cell lines, and **(B)** Vero E6 and Vero E6-TMPRSS2 cell lines. Growth curves showing the release of viral genome into the medium of cells incubated for 1 h with a 0.001 MOI of both VOCs. The ΔCt were reported as the mean values ± SD, **p < 0.01, ***p < 0.001 and ****p < 0.0001. Ct: threshold cycle.

We performed infection kinetics assays to evaluate if the two viral variants were characterized by different replication abilities. For the first evaluation, we used human epithelial colorectal adenocarcinoma (Caco-2) and human lung cancer (Calu-3) cells to be more consistent with the site of primary infection of the virus. Viral RNA was purified, reverse transcribed, and detected using Real-Time PCR with N-specific primers at different time points to assess infection kinetics. The results showed that XBB.1 was slower than XBB.1.5 at 24- and 48-hours post-infection (hpi) in both cell lines (p < 0.001 in Calu-3, p < 0.01 in Caco-2 cells, **Figure 1A**). No differences were observed at 72 hpi, likely because any initial variations had leveled out by this time point. To verify that the differences between XBB.1 and XBB.1.5 were restricted to the S protein, a pseudovirus entry assay was conducted. The results indicated that XBB.1.5 entered Calu-3 cells more rapidly than XBB.1 (p < 0.01, **Supplementary Figure S1**).

However, the infection might be affected by the innate immunity of the selected cell lines. To address this and focus solely on the molecular characteristics of the two viral variants, the kinetic experiment was repeated using Vero E6 cells. This cell line, which lacks genes encoding type I interferons (IFN), is permissive to SARS-CoV-2 infection due to its expression of ACE2. Both wild-type cells and those stably expressing TMPRSS2 were used to investigate the potential role of the endocytosis-dependent entry route, as opposed to direct fusion at the cell surface. Our results confirmed the differences observed at 24 and 48 hpi, but only in the absence of TMPRSS2 expression (p < 0.01 and p < 0.001 in Vero E6, **Figure 1B**), likely because overexpression of the serine protease in the engineered cells (p < 0.0001, **Supplementary Figure S2**) favored the entry by direct plasma membrane fusion, which, being faster, masked the differences previously observed. This increase was expected, as cell lines modified to stably express heterologous proteins typically undergo optimization of transcription, translation, folding, and secretion processes, resulting in much higher protein yields compared to non-engineered cells.

**Figure 2:**
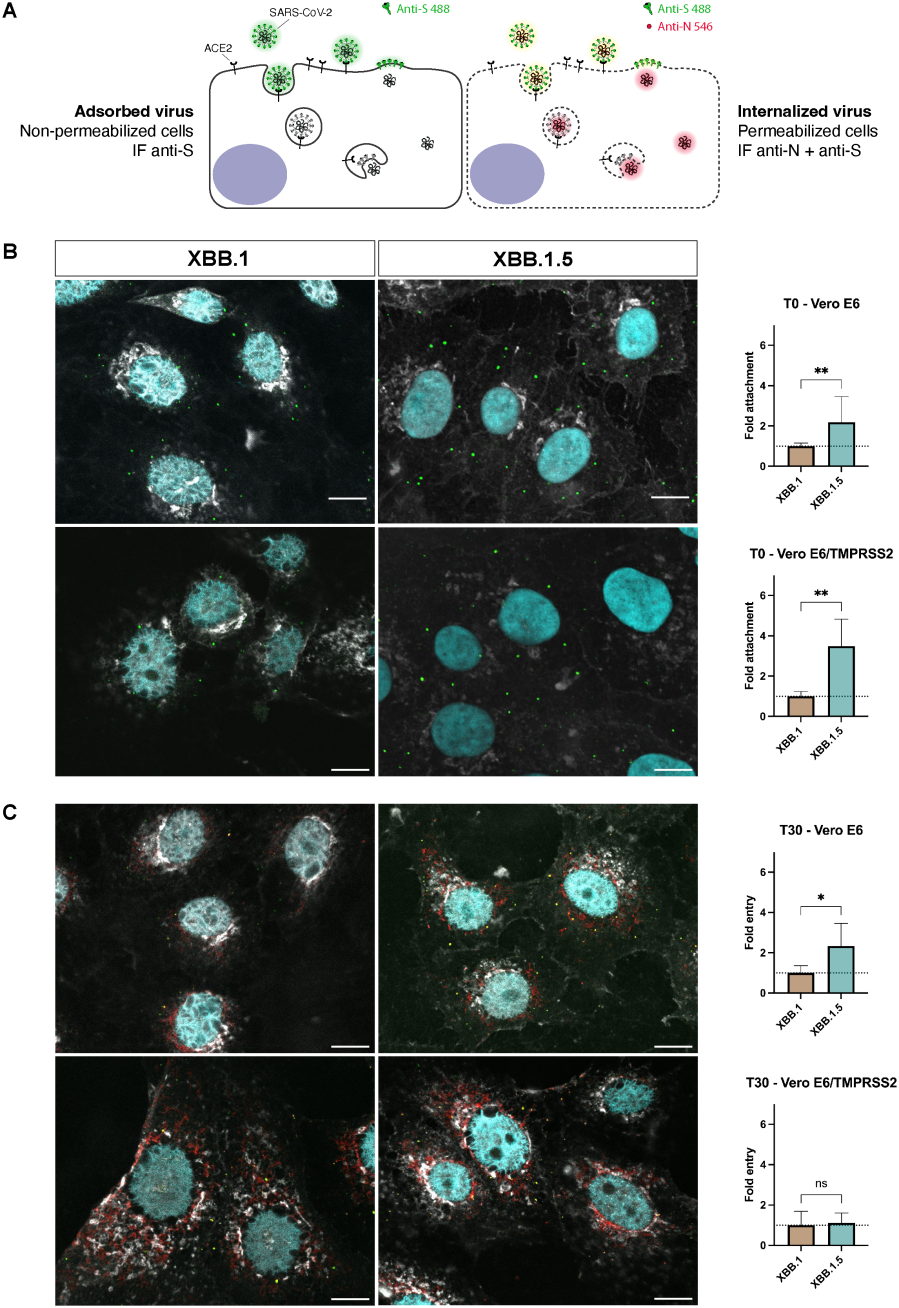
Differences between XBB.1 and XBB.1.5 attachment and entry processes. **(A)** Schematic representation of the two-step antibody staining procedure performed with antibodies against S (green) and N (red) to distinguish adsorbed and internalized virus particles. **(B)** The number of virus particles adsorbed to the cell surface at T0 identified by S protein events (green signal) was relatively quantified using XBB.1 as reference. Mean + SD, **p < 0.01 (n = 5, 150 nuclei). **(C)** Levels of N protein 30 minutes (T30) after the virus adsorption at 4°C measured as integrated density values normalized on the number of nuclei (cyan signal) and relatively quantified using XBB.1 as standard. Mean + SD, *p < 0.05 (n = 5, 150 nuclei). 60x magnification, scale bar 10 μm. One representative ROI of each experimental condition is reported. ns, Not significant.

XBB.1 and XBB.1.5 spike proteins differ just for one aminoacidic substitution (**Supplementary Figure S3**). As kinetic experiments did not allow the fine dissection of the initial steps of the entry process where such a minimal aspect can make a difference (< 6hpi), we performed an entry assay using confocal microscopy quantification. To determine whether a single mutation enhances the binding capacity of the S protein to the cell surface and/or accelerates viral entry, we measured the number of viral particles on the target cell surface under two conditions. In the first condition, virus particles were added to pre-cooled cell monolayers and incubated for 30 minutes at 4°C, a temperature that inhibits membrane fusion and prevents virus entry. In the second protocol, an additional incubation at 37°C was performed, allowing surface-bound viral particles to enter the cells synchronously, as the S protein facilitates membrane fusion at this temperature. A dual-staining technique was employed, enabling us to distinguish between viral particles that remained adsorbed on the cell surface and those that had been internalized (**Figure 2A**). Both experimental settings showed a higher attachment of XBB.1.5 virions to both cell lines (p < 0.01, **Figure 2B**), and a slower entry kinetics of XBB.1 in Vero E6 (p < 0.05, **Figure 2C**) compared to Vero E6-TMPRSS2 cells. These findings confirmed that a single S mutation enhances XBB.1.5 infection by influencing the earliest molecular mechanisms of viral entry. We ensured that the differences observed were not attributable to the preparation of the viral stocks by quantifying the number of particles in the dilutions used, and we found no disparities (**Supplementary Figure S4**).

### 3.2 The S mutation promotes its fusion activity

**Figure 3:**
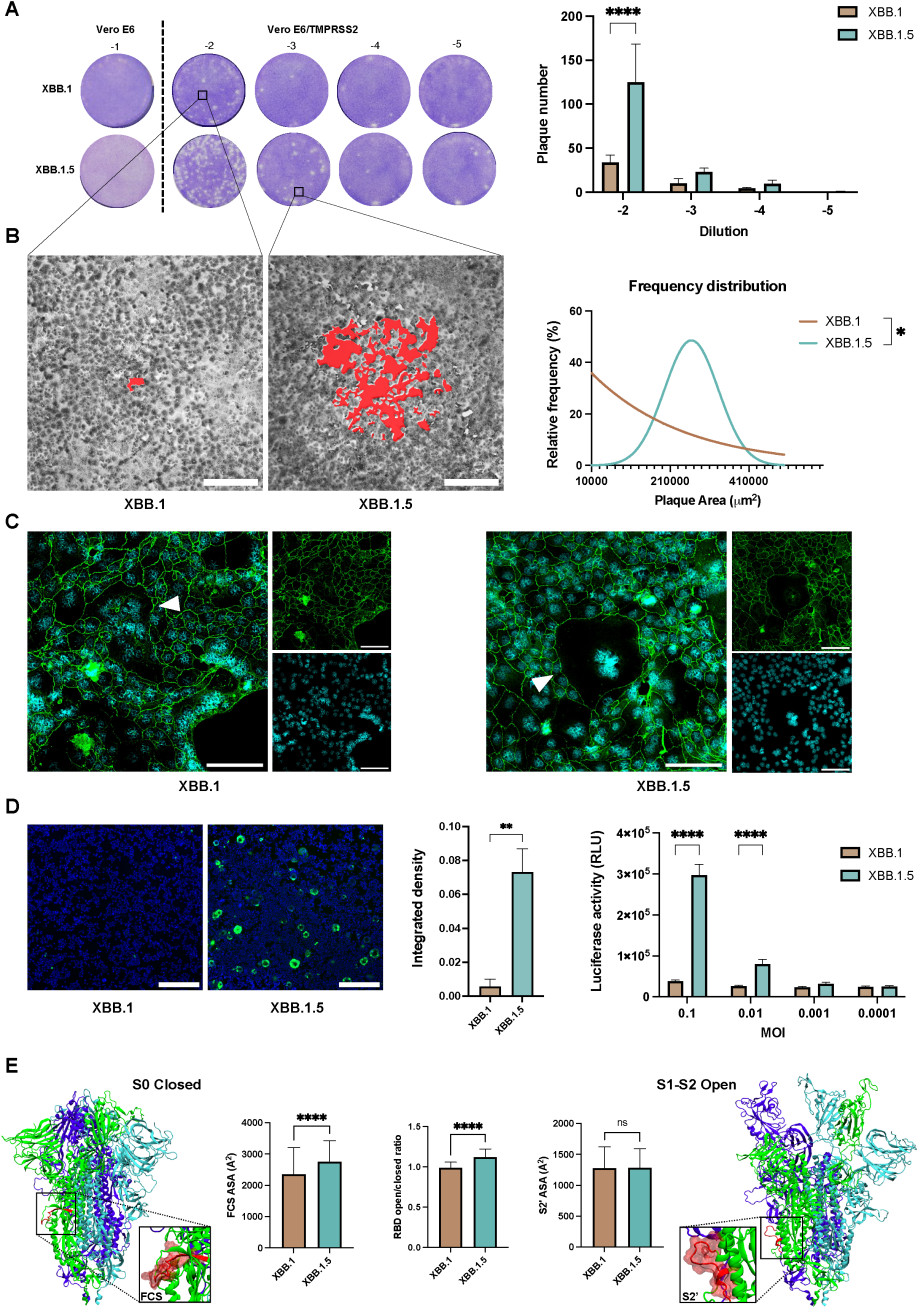
Cell-to-cell transmission mechanism evaluation. **(A)** Plaque assay performed on Vero E6 and Vero E6-TMPRSS2 to assess cell-to-cell spreading differences within the XBB.1 and XBB.1.5 variants 46 hpi. Bars show the number of plaques obtained on Vero E6-TMPRSS2 cells with 10-fold dilutions of either XBB.1 or XBB.1.5. Means with SD are reported (n = 2), ****p < 0.0001. **(B)** Evaluation of the area of plaques resulted from XBB.1 and XBB.1.5 infections, highlighted in red in the two representative magnifications, and reported as relative frequency distribution. *p < 0.05. 5x magnification, scale bar 200 μm. **(C)** Formation of syncytia 72 h post XBB.1 or XBB.1.5 infection of Calu-3 cells (white triangles). Nuclei in cyan, cell contours in green using a-ZO1 staining. Scale bars, 100 μm. **(D)** Quantification of the fusogenic events detected 24 hpi (0.001 MOI) in a co-culture of 293T-ACE2.TMPRSS2 cells transfected with the cell fusion reporting system. Representative images (n = 5) are reported, 5x magnification, scale bar 200 μm. Syncytia formation was quantified by integrated density of the mNeonGreen protein fluorescent signal normalized to the integrated density of cellular nuclear staining. Means + SD are reported, **p < 0.01. Fusion efficiency was measured 24h after infection with different MOIs as luciferase activity (Relative luminescence units, RLU). Means + SD are reported, ****p < 0.0001. **(E)** Dynamics and energetic features of XBB.1 and XBB.1.5 spike representative of FCS and S2’ spike priming and S1 release. ns, Not significant.

The SARS-CoV-2 S protein also facilitates cell-to-cell spread, allowing viral progeny from an infected cell to be directly transferred to neighboring cells. This occurs when TMPRSS2 is expressed on the cell surface, enabling the S protein to promote membrane fusion between adjacent cells (28). This direct transfer accelerates the infection process compared to the slower route of viral release into the extracellular space followed by reinfection. In the plaque assay, a semisolid medium is applied to the cell monolayer immediately after virus adsorption, blocking the virus cell-free route of infection and allowing only direct cell-to-cell spread. When a plaque assay was performed with serial dilutions of the viral stocks, the results revealed a significant difference in both the number (p < 0.0001, **Figure 3A**) and the size of the plaques (p < 0.05, **Figure 3B**) between the two viral variants, highlighting the impact of this direct spread mechanism.

This demonstrated that both variants could utilize the diffusion mechanism when TMPRSS2 is present on the cell surface. To further explore this, we assessed whether the same phenomenon occurred in lung epithelial cells that endogenously express this surface serine protease. Confocal imaging revealed the presence of multinucleated cells post-infection, with more pronounced effects observed when using the XBB.1.5 variant (**Figure 3C**).

To better quantify fusogenic events, we used a reporting system that Huang et al. developed by fusing a pair of split NanoLuc luciferase, LgBiT (1–159 amino acids) and SmBiT (160–171 amino acids), and split mNeonGreen protein, NG (1–173 amino acids) and CG (174–236 amino acids), with a pair of interacting leucine zippers (bFos-bJun) (28). When cells fuse, both NanoLuc luciferase and mNeonGreen proteins are reconstituted to ensure the quantitative detection of cell-fusion efficiency. When the four fusion proteins were transfected separately (SJNG, NGJS, LFCG, or CGFL), no luciferase activity was detected, confirming that the reporting system has low background signals. When a pair of fusion proteins was co-expressed, the interaction between bFos and bJun restored the NanoLuc luciferase activity and mNeonGreen signal to different extents, and the combination of NGJS and CGFL produced the optimum results (p < 0.0001, **Supplementary Figure S5**). Thus, we infected a co-culture of 293T-ACE2.TMPRSS2 cells, pre-transfected with NGJS and CGFL, using XBB.1 and XBB.1.5 variants. The results aligned with plaque assay data, indicating that the XBB.1.5 variant is extremely effective in inducing syncytia formation, as confirmed by both fluorescent signal and luciferase activity quantification (p < 0.01 and p < 0.0001, **Figure 3D**). To further confirm the impact of the single S mutation on cell-cell fusion, we conducted the same assay with cells transfected with either the XBB.1 or XBB.1.5 S protein alone. These findings aligned with those observed in infected cells (p < 0.05, **Supplementary Figure S6**).

To provide a mechanistic explanation for XBB.1.5 higher sincytiation, we evaluated *in silico* three spike parameters representative of molecular events strictly necessary for fusion: furin and TMPRSS2 cleavage, and RBD opening. Indeed, processing at the FCS is necessary to allow S1 release upon RBD opening and S2’ cleavage is required to expose the fusion peptide. Compared to XBB.1, XBB.1.5 spike presents a significantly more exposed FCS – that directly correlates with its processing rate – and higher propensity to open the RBDs, while the S2’ site cleaved by TMPRSS2 is equally exposed in the two variants (**Figure 3E**). Thus, the single RBD mutation differentiating XBB.1 and XBB.1.5 has local and allosteric effects – affecting RBD opening and FCS exposure, respectively – that promote spike fusion activity by increasing the S1-S2 dissociation rate but do not affect the fusion peptide exposure.

### 3.3 The S mutation inhibits sera from neutralizing cell-to-cell spread

We assessed whether the observed differences in infection kinetics and spread between the two SARS-CoV-2 variants could influence antibody neutralization activity. First, twenty-nine sera from recovered healthcare professionals (**Supplementary Table S1**) were tested for their ability to block virus entry into Vero E6 cells. We chose those cells as they represent the gold standard for serum neutralizing (NT) activity testing and allowed us to assess if the antibodies directly hampered the binding between S and the cellular receptor ACE. Its expression level was comparable to what was detected in Caco-2 and Calu-3, but different from 293T-ACE2.TMPRSS2 cells, as expected (p < 0.0001, **Supplementary Figure S7A**).

**Figure 4:**
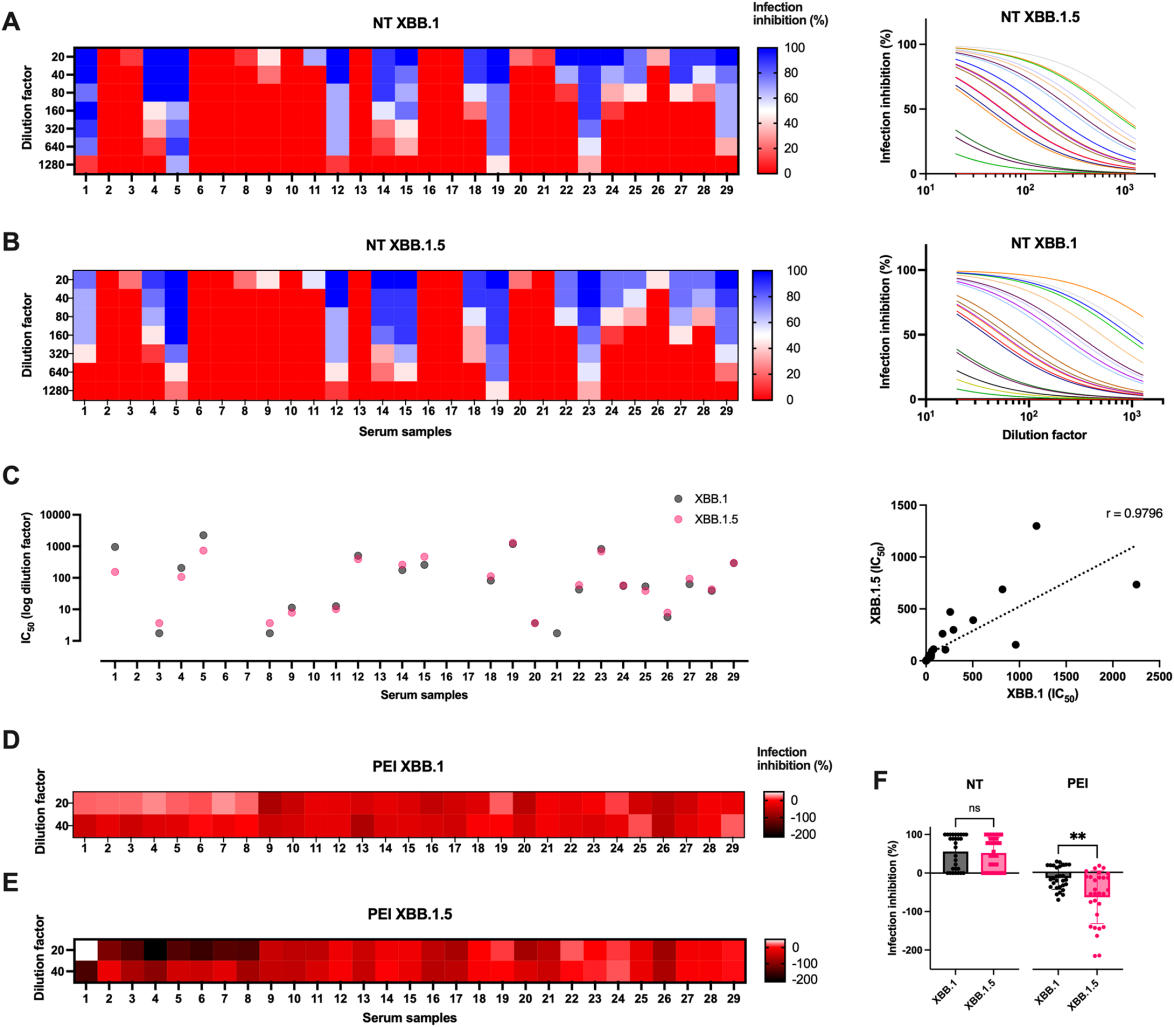
Sera biological activity against virus infection *in vitro*. **(A)** Serial dilutions of serum samples from healthcare professionals were tested in microneutralization (NT) experiments against XBB.1 and **(B)** XBB.1.5 infections (0.01 MOI). Data are reported as infection inhibition (%) normalized on the infection control condition. **(C)** Graphs showing the IC_50_ values expressed as dilution factor of the tested sera against the two infections, and their Spearman’s correlation analysis (dotted line, r = 0.9796, p < 0.0001). **(D)** Sera were tested for their post-entry inhibition (PEI) of XBB.1 and **(E)** XBB.1.5 infection (0.01 MOI), measuring the fusogenic events observed when using the two highest dilutions (1:20 and 1:40). Data are reported as infection inhibition (%) normalized on the infection control condition. **(F)** The antiviral activity of sera against the two variants was compared as NT and PEI activity observed at 1:20 sera dilution. Means + SD are reported, **p < 0.01. ns, Not significant.

NT assay showed that almost half of the selected samples were able to prevent both virus infections (**Figures 4A, B**; **Supplementary Tables S2, S3**). Moreover, the data showed the presence of a correlation between the IC_50_ obtained from the samples against the two virus variants, indicating that the same sera neutralized both variants (r = 0.9796, p < 0.0001, **Figure 4C**).

However, since the greatest difference between the two variants was observed in their cell-to-cell spreading ability, the sera were tested for their ability to prevent syncytia formation using the fusogenic reporting system. 293T-ACE2.TMPRSS2 cells resulted in our preferred choice as they express TMPRSS2 like untransfected cell lines, compared to engineered Vero E6 (p < 0.001, **Supplementary Figure S7B**). Therefore, the post-entry inhibition (PEI) assay was performed to evaluate the ability of sera to impair virus spreading even after its entry into the target cells. Briefly, cocultures of cells pre-transfected with NGJS and CGFL were infected, and, after washing cell-free virus particles, culture medium containing different dilutions of the sera was added. This method allowed us to appreciate virus spreading into adjacent cells via the cell-to-cell mechanism of infection. In contrast to the behavior of the same sera tested at the same dilutions for their neutralization capability, almost all samples favored cell-to-cell transmission. This resulted in negative infection inhibition values, as we observed a significantly stronger cytopathic effect when sera were added to the infected cells compared to the control condition that contained only the virus (**Figures 4D, E**). Yet, there seemed to be a difference between what we observed against the two variants, and therefore we compared the activity of the sera between the two infections in the two experimental settings. The analysis confirmed that the sera activity was comparable against the two SARS-CoV-2 infections in terms of neutralization of the cell-free virus, but only XBB.1 cell-to-cell spreading was slightly hindered, in agreement with what was observed on the more marked fusogenic capacity of the variant XBB.1.5 (p < 0.01, **Figure 4F**).

**Figure 5:**
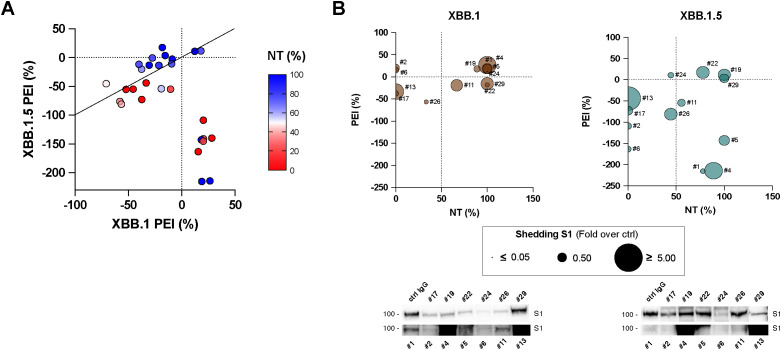
Correlation analyses. **(A)** Analysis conducted on the infection inhibition capability of all the tested sera (1:20 dilution) and their post-entry inhibition (PEI) against XBB.1 (*x*-axes) and XBB.1.5 (*y*-axes) variants. Serum samples are represented by their NT activity, Pearson correlation analysis and line of identity are shown. **(B)** A selected cohort of sera (#1, #2, #4, #5, #6, #11, #13, #17, #19, #22, #24, #26, #29) was tested for their ability to mediate S1 shedding on cells transfected with XBB.1 or XBB.1.5 recombinant S protein, and results were correlated with NT and PEI data. Immunoblots showing shedded S1 subunits are reported.

We conducted further investigations to determine if a correlation existed between NT and PEI activity for each tested serum at their lower dilution (1:20). Our analysis revealed a subset of sera demonstrating correlated activity between the experimental data against both viral variants (**Figure 5A**). Specifically, the line of identity indicates which sera exhibit a comparable effect against the two variants in PEI assays, with the ability to restrain cell-to-cell spreading closely linked to the observed neutralizing activity. However, it is noteworthy that a subset of both neutralizing and non-neutralizing sera deviates from this pattern, exhibiting PEI inhibition of XBB.1 while facilitating direct cell-to-cell transmission of XBB.1.5 infection (r = -0.5, p < 0.01, **Figures 5A**; **Supplementary Figure S8A**).

We hypothesized that sera might facilitate this process due to the binding to the S expressed on the surface of the infected cells and inducing rapid shedding of the S1 subunit. This action likely mimics the role of ACE2 during infection, thereby promoting cell-cell transmission of the SARS-CoV-2 virus (29). Indeed, during biosynthesis and maturation in the infected cell, the S protein is cleaved by furin or furin-like proprotein convertases in the Golgi apparatus into the S1 and S2 subunits, which remain non-covalently associated. The transition of the S protein into its fusion-ready state involves the separation of the S1 subunit, which carries the receptor-binding domains, from the membrane-bound S2 subunit. Binding to ACE2 or a decoy antibody is sufficient to induce this process (30). To determine this aspect, we collected supernatants from XBB.1 and XBB.1.5 S-transfected cells stimulated with a selected cohort of sera (n = 13) and quantified the presence cleaved spike protein products using immunoblots. We detected different release of cleaved S1 subunits into the cell supernatants in all experimental conditions, and results were correlated with NT and PEI data (**Figure 5B**; **Supplementary Figure S9**). The findings showed that sera with a higher S1 shedding rate did not align with either the neutralizing or post-entry inhibiting ones. There was actually no correlation between the biological activity of the selected sera against the two infections, except for their neutralizing activity against both viral variants (r = 0.809, p < 0.01, **Supplementary Figure S8B**).

## 4 Discussion

The emergence of two VOCs differing for just a single mutation in the S protein represented an important evolution event that allowed us to understand how even a single amino acid modification can impact the epidemiological situation. In our study, we examined the complete life cycle of SARS-CoV-2 variants XBB.1 and XBB.1.5, which differ by the S486P mutation that has persisted in the most recent XEC variant (31), and the E148G mutation in nonstructural protein 1 (Nsp1), which has a neutral effect on viral replication as it has never become fixed in the course of viral evolution (32). The aim was to determine if there were any variations in terms of entry, replication, or spreading efficiency.

First, the infection kinetics analyzed at different time points showed how the XBB.1.5 variant was faster in two cell lines which should mimic *in vitro* the characteristics of the infection sites in the respiratory and digestive systems. However, as the immune recognition of the host cell used could have influenced the result, we repeated the experiment with a defective cell line, Vero E6. This aspect makes them unable to mount an efficient innate immune response to virus infection but does not affect their permissiveness to SARS-CoV-2 infection, thus allowing to better focus on the molecular mechanisms involved in the virus life cycle. Results confirmed the slower kinetic of the XBB.1 variant, which is, therefore, more linked to the S binding and fusion properties within the endosomal membrane than to its immune recognition. Yet, those cells lack TMPRSS2, and if a virus–ACE2 complex does not encounter this plasma membrane-associated protease, it is internalized via clathrin-mediated endocytosis into the endolysosomes, where S2′ cleavage is performed by cathepsins (33). Therefore, to expand the characterization of the infection kinetics, we also used Vero E6-TMPRSS2 cells to allow direct envelope fusion at the plasma membrane. In this case, we no longer saw significant differences between the two infections. The cause is probably that the two entry pathways involve different molecular processes, which result in different speeds at which the viral genome is released into the cytosol. The engineered cells overexpress TMPRSS2 on the cell surface, polarizing the virus entry towards the fastest route and not allowing us to appreciate minimal differences between the two variants due to the method used for the detection.

The experimental protocol used has the important limitation as it does not allow a detailed study of the early stages of infection, such as virus attachment and entry. This is because it only detects the viral progeny, which could be influenced by different behaviors in these processes. To address this limitation, we employed laser point-scanning confocal microscopy to investigate whether the varying speed of infection of the SARS-CoV-2 variants was due to their ability to enter the target cell. Our infection protocol and staining procedures were optimized to achieve simultaneous viral entry and distinct fluorescent signals for adsorbed and internalized viral particles. Our initial investigation focused on whether the reported enhanced binding affinity of the XBB.1.5 spike protein affects viral replication kinetics from the early stages, allowing the virus to bind to the cell surface without being internalized. Our results confirmed the literature findings (7, 8, 11, 12, 34), showing that XBB.1.5 indeed demonstrates superior binding affinity. Subsequently, quantification of the N signal within Vero E6 cells after allowing virus internalization revealed a significant difference in favor of the XBB.1.5 virus, suggesting that it may more effectively exploit the endocytosis pathway. These data are consistent with previous experiments using lentivirus-based pseudoviruses, which demonstrated approximately a 3-fold increase in infectivity of XBB.1.5 compared to XBB.1 (12). Once more, no differences were measured in engineered Vero E6 cells, confirming our speculation about the rate of entry via direct fusion at the cell surface. The results indicated that there was a difference in the ability of the two viruses to bind to Vero E6 and Vero E6-TMPRSS2 cells. This is not surprising as the S of the viruses are almost identical except for the RBD, and virus adsorption depends on ACE2 recognition. However, the ACE receptor mainly facilitates virus entry rather than attachment. The attachment process involves additional factors such as coreceptors, surface charges, and S2’ processing (35). Importantly, differences between the two variants were observed during the evaluation of virus entry using Vero E6 cells. Our results indicate that when entry through endocytosis is the only viable pathway, XBB.1.5 shows significantly enhanced entry, which supports the findings from kinetic experiments.

Another aspect that we wanted to explore was if the S mutation affects an additional process in which the S fusogenic properties are involved, cell-to-cell transmission. Indeed, enveloped viruses spread in cell cultures and tissues via two routes: cell-free particles and cell-to-cell contact (36). Additionally, cell-to-cell transmission evades antibody neutralization, leading to efficient virus spread and pathogenesis, as has been shown for HIV, HCV, and HSV (37–40). Studies have demonstrated that SARS-CoV-2 spike-mediated cell fusion results in the creation of abnormal and multinucleated cells not only in the lungs of patients but also in human small intestinal enterocytes, as well as neuronal and glial fusion *in vitro* (41–43). To date, it has been indirectly demonstrated that SARS-CoV-2 can spread through cell-cell contacts by using recombinant S transiently expressed on transfected cells, or lentiviral pseudotyped viruses (13, 27, 43, 44). Thus, we performed a plaque assay using Vero E6-TMPRSS2 cells to evaluate the syncitiation capability of the two variants. Surprisingly, infection with XBB.1.5 results in a significantly higher number and larger size of plaques compared to XBB.1, despite the lack of any significant difference in cell-free virus entry in the same cell line in the previous experiments. This result was confirmed using a reporting system that exclusively measures spike fusion efficiency. It is consistent with published literature showing that the S recombinant proteins of BQ.1.1 and XBB.1.5 promote cell-to-cell fusion more efficiently than those of earlier Omicron variants (13, 27).

Since the emergence of SARS-CoV-2, multiple vaccines and monoclonal antibodies were developed and approved for clinical use. All vaccines encode for or contain the spike protein, as it is the sole mediator of SARS-CoV-2 entry into host cells and target of neutralizing antibodies. Thus, we evaluated if the S486P mutation differentiating XBB.1 and XBB.1.5 has an impact on their recognition by sera from recently infected healthcare professionals. In detail, the neutralization assay indicated that about half of the samples contained antibodies able to block the cell-free virus infection of both variants. Thanks to the heterogeneity of the selected cohort, it was possible to highlight how the sera capable of preventing XBB.1 infection were the same showing activity against XBB.1.5. We hypothesized that, when only cell-free viral particles are present, the S486P mutation is not sufficient to hamper the activity of anti-RBD neutralizing antibodies, and the greater speed observed in the 30-minute entry assays of XBB.1.5 over XBB.1 does not affect serum neutralization.

The previous results showed that XBB.1.5 can spread more effectively from one cell to adjacent cells compared to XBB.1. We tested whether sera were able to stop this type of transmission. Interestingly, the global behavior of the sera in inhibiting the direct spreading after virus entry diverged from the overall trend observed in neutralization experiments. Using a system that exclusively quantifies cell-to-cell spread, without considering cell-free virus transmission, we found that nearly all sera failed to consistently inhibit syncytia formation. The little activity observed was statistically more effective against the XBB.1 subvariant. This behavior has never been described before for humoral responses directed against SARS-CoV-2, both naturally elicited or induced by vaccination, but has been verified for HCMV and HSV infections (40, 45). Published data only reported that a small batch of serum samples from COVID-19 patients (n = 5) as well as pooled sera from mRNA vaccinees were not able to prevent S-mediated membrane fusion when tested on cells transfected with S from various variants (WT, D614G, Alpha, and Beta) (46). The same method was used to demonstrate that mouse monoclonal antibodies can reduce cell-cell fusion caused by WT, Alpha, Beta, Gamma, Kappa, Delta, and Omicron S proteins, dependent or independent of the ACE2 receptor (47). Even if an infection model was not used, these data validate that antibodies may have the ability to block this mode of transmission. However, those molecules appear very poorly represented within the total antibody response elicited by SARS-CoV-2 infection or vaccination. On the contrary, we observed that most tested sera promote syncitiation in a dose-dependent manner, particularly when XBB.1.5 was used. This phenomenon has recently been elucidated by the interaction of Class I antibodies with the receptor binding motif (RBM) within the RBD, the region directly taking contact with ACE2. Specifically, when the SARS-CoV-2 spike protein undergoes cleavage at the S1/S2 cleavage site and is expressed on the infected cell membrane, the binding of these antibodies triggers rapid shedding of the S1 subunit at the cell surface, likely mimicking the function of ACE2 in infection. This shedding event allows TMPRSS2 to process the S2’ cleavage site, thereby exposing the fusion peptide in spike proteins expressed on the cell membrane and consequently promoting cell-cell transmission of the SARS-CoV-2 virus (29). However, we showed that the sera characterized by a higher S1 shedding did not cluster among the neutralizing ones, nor among those that facilitate the spread of infection. We therefore demonstrate for the first time that this event is not correlated with either the neutralizing activity towards cell-free virus particles, or in promoting cell-cell transmission mechanisms.

As none of the sera endowed with high neutralizing capacity showed superior activity to non-neutralizing samples in preventing cell-to-cell, we can conclude that the antibodies capable of blocking the entry of cell-free particles must be different from those that hinder the fusion of cell membranes, especially in terms of epitope localization. Indeed, the work by Reuter et al. assumes that S fusion activity could be inhibited by antibodies directed against its N-terminal domain (NTD) but not by antibodies targeting its RBD (47). Last, as cell-cell fusion is of major importance for the dissemination of the virus, we hypothesized that the emergence of a VOC must be related also to an increased ability to disseminate through this molecular mechanism. Indeed, we observed that a single mutation allowed XBB.1.5 to exploit this spreading route more quickly and efficiently than XBB.1. Consequently, not only were all the sera tested entirely incapable of slowing it down, but conversely, they significantly promoted it.

In conclusion, our results stress the importance of finely characterize all the molecular processes in which the S protein is involved to reliably predict the impact of future SARS-CoV-2 variants, provide the rationale for new and more effective therapies.

## Data Availability

The datasets presented in this study can be found in online repositories. The names of the repository/repositories and accession number(s) can be found below: GISAID (https://gisaid.org/), the ID of the uploaded data are: XBB.1 (GISAID accession ID: EPI_ISL_16526278) and XBB.1.5 (GISAID accession ID: EPI_ISL_18537906).
