# Supplementary material for: Single spike mutation differentiating XBB.1 and XBB.1.5 enhances SARS-CoV-2 cell-to-cell transmission and facilitates serum-mediated enhancement
Source: Front Immunol. 2024 Nov 27;15:1501200. doi: 10.3389/fimmu.2024.1501200 (PMC11631925; doi:10.3389/fimmu.2024.1501200)
Supplement: Supplementary file 1 [file DataSheet1.docx]

**Supplementary materials**

**
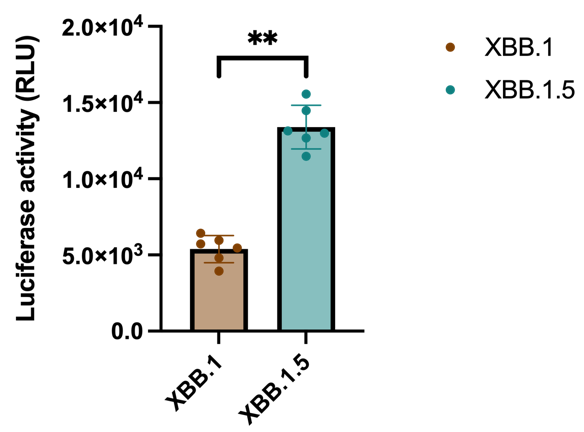
**

**Figure S1. Pseudovirus infection.** Pseudovirus particles carrying XBB.1 or XBB.1.5 S were used to infect Calu-3 cells at equal doses, and luciferase activity (Relative luminescence units, RLU) was detected after incubation at 37°C for 72 h. Data from six replicates are shown as the mean ± SD, ** p < 0.01.

**
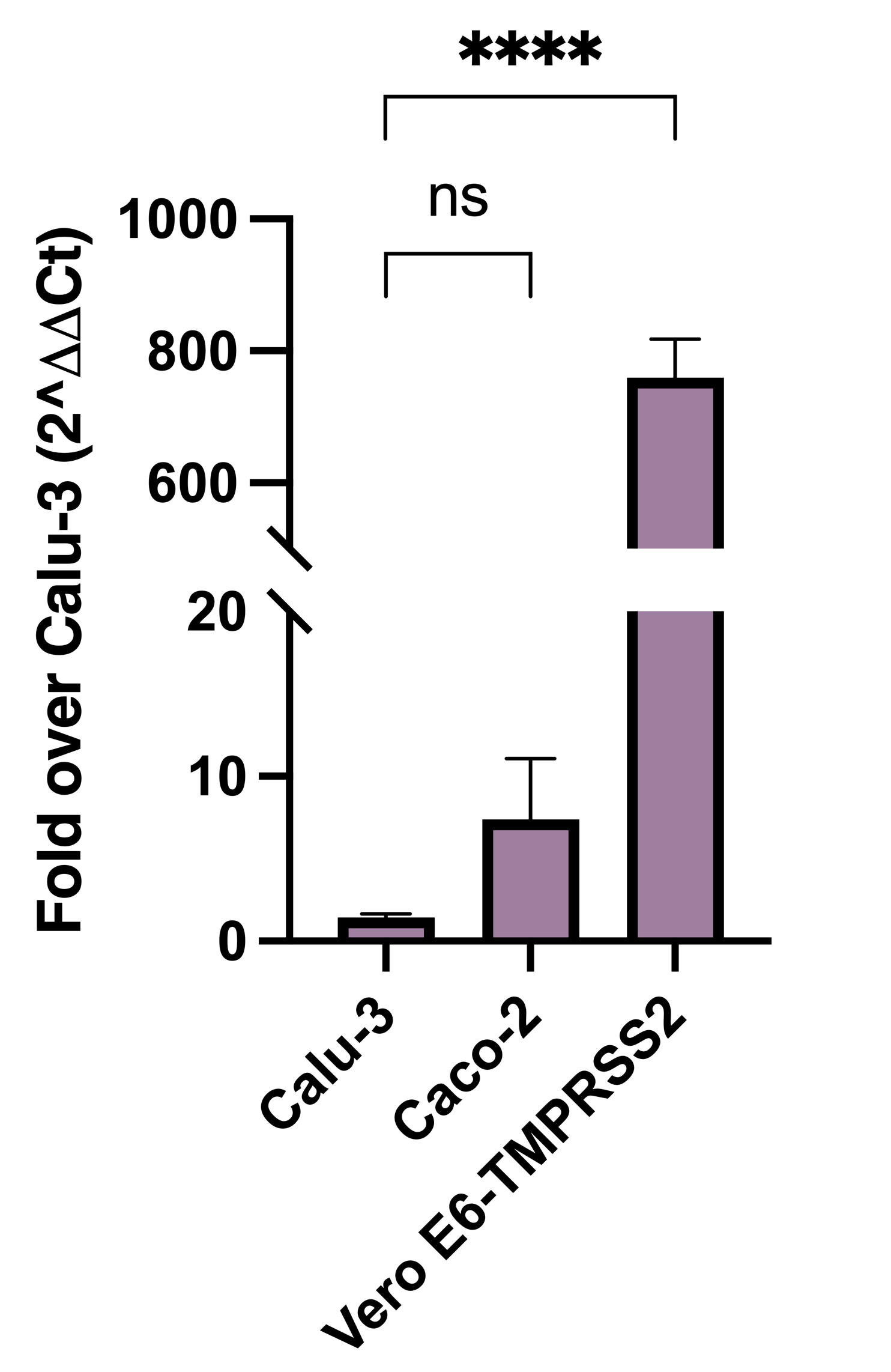
**

**Figure S2. Expression levels of TMPRSS2 in the different cell lines.** Gene expression analysis was performed on Calu-3, Caco-2, and Vero E6-TMPRSS2 cell lines. Each condition was tested in triplicate**.** Mean + SD are reported, **** p < 0.0001.

**A**

| Gene | Only XBB.1 | Shared | Only XBB.1.5 |
| --- | --- | --- | --- |
| E | - | T9I, T11A | - |
| M | - | Q19E, A63T | - |
| N | - | P13L,E31-,R32-,S33-,R203K,G204R,S413R | - |
| ORF1a | - | K47R,S135R,T842I,G1307S,L3027F,T3090I, L3201F,  T3255I, P3395H, S3675-, G3676-, F3677- | - |
| ORF1b | - | P314L, G662S, S959P, R1315C, I1566V, T2163I | - |
| ORF3a | - | T223I | - |
| ORF6 | - | D61L | - |
| ORF7a | - | 0 | - |
| ORF7b | - | 0 | - |
| ORF8 | - | G8* | - |
| ORF9b | - | P10S, E27-, N28-, A29- | - |
| S | **F486S** | T19I, L24-, P25-, P26-, A27S, V83A, G142D, Y144-, H146Q, Q183E, V213E, G252V, G339H, R346T, L368I, S371F, S373P, S375F, T376A, D405N, R408S, K417N, N440K, V445P, G446S, N460K, S477N, T478K, E484A, F490S, Q498R, N501Y, Y505H, D614G, H655Y, N679K, P681H, N764K, D796Y, Q954H, N969K | **F486P** |

**
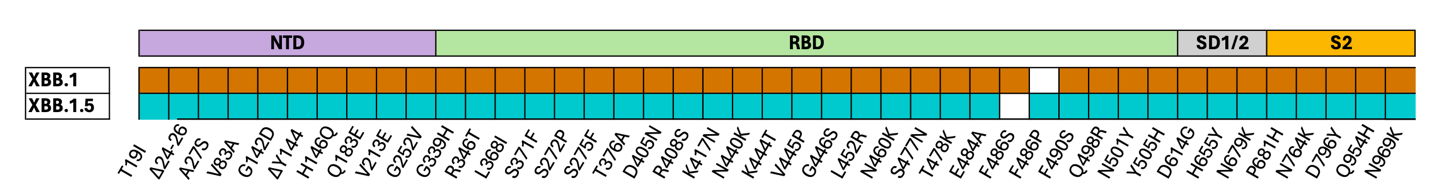
**

**B**

**Figure S3. Amino acid changes between XBB.1 and XBB.1.5 variants. A)** Analysis of the distribution of amino acid changes in the viral genes using the Nextclade reference sequences of the two variants **B)** Schematic view of S mutations in SARS-CoV-2 variants evaluated in this study. SD1/2, subdomains 1 and 2.

**Figure S4. Quantification of viral particles.** XBB.1 and XBB.1.5 stock dilutions (1 MOI) used in infection kinetics and entry experiments were quantified through Real-time PCR. Cycle thresholds (Ct) were reported as the mean values + SD.

**
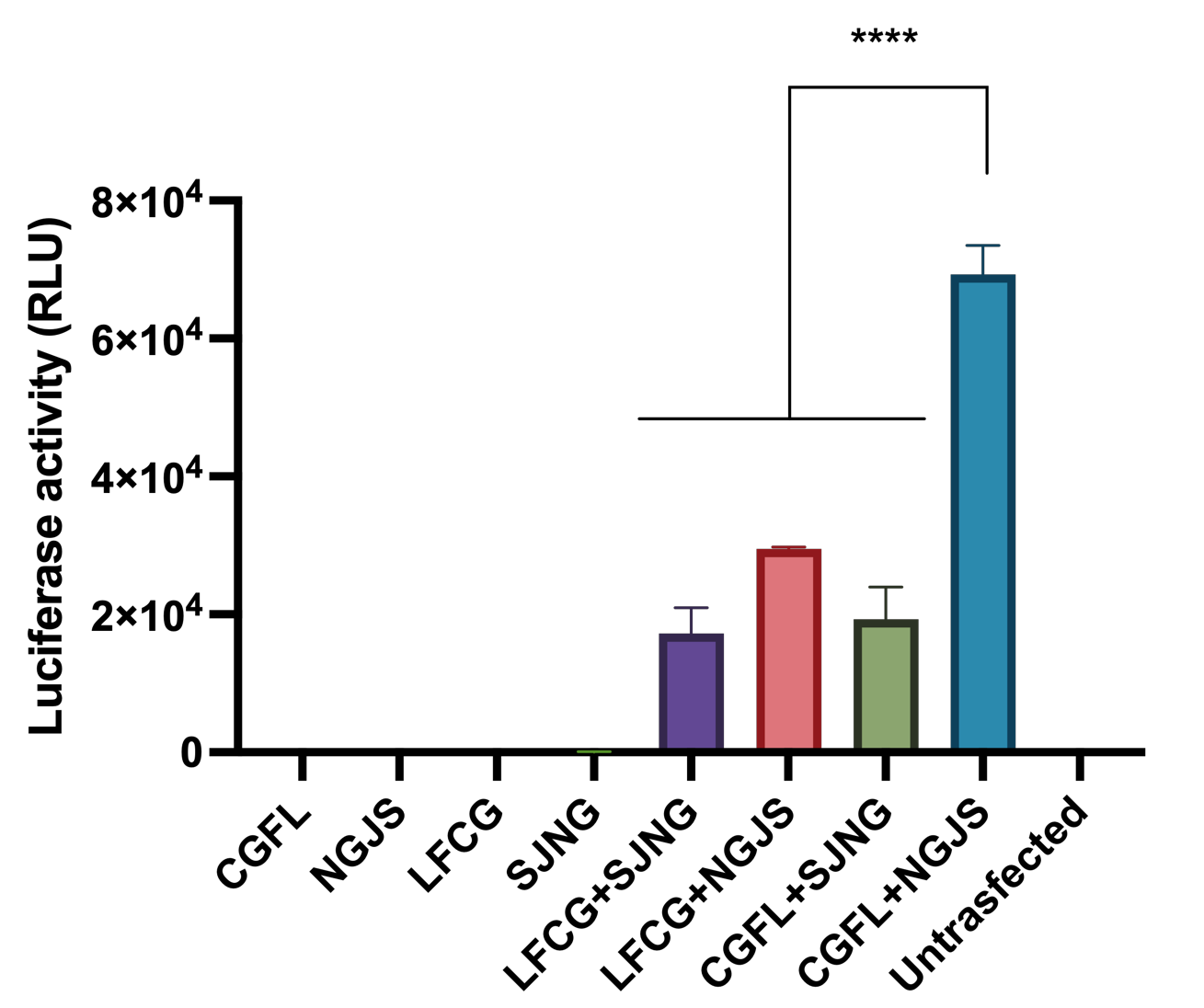
**

**Figure S5. Validation of the reporting system for measurement of SARS-CoV-2 spike fusion efficiency.** Four different recombining proteins comprising split mNeonGreen (NGJS and SJNG) and split NanoLuc (LFCG and CGFL) were fused with different ends of bJun or bFos. The luciferase activities (Relative luminescence units, RLU) of these four different recombining proteins were detected after transfecting or co-transfecting their plasmids into 293T-ACE2.TMPRSS2 cells for 72 h. Each condition was tested in sixfold**.** Mean + SD, **** p < 0.0001.

**Figure S6. Spike-mediated cell fusion evaluation.** Quantification of the fusogenic events induced by XBB.1 or XBB.1.5 S protein in a co-culture of 293T-ACE2.TMPRSS2 cells transfected with the cell fusion reporting system. Fusion efficiency was measured 24h after transfection as luciferase activity (Relative luminescence units, RLU). Means + SD, * p < 0.05.


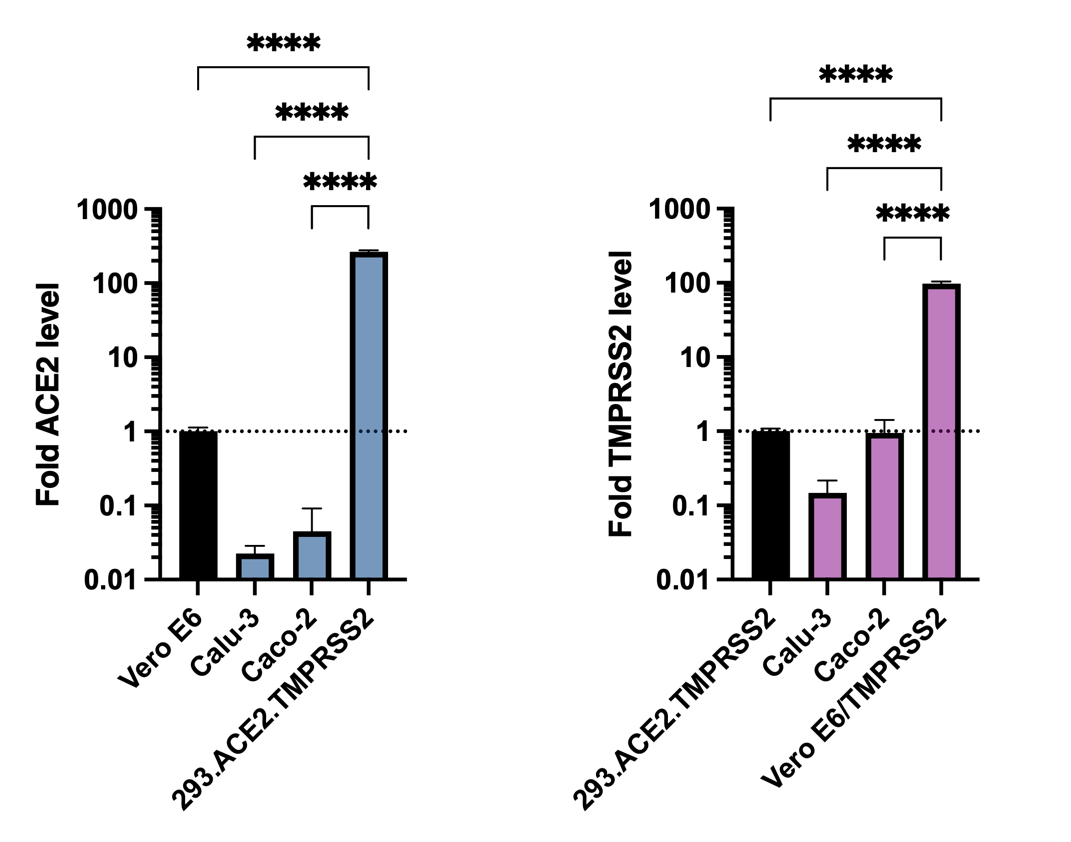


**A**

**B**

**Figure S7. Different expression levels of (A) ACE2 and (B) TMPRSS2.** Gene expression was analyzed using Vero E6 as reference for ACE2, and 293T-ACE2.TMPRSS2 for TMPRSS2 (dotted lines and black histograms). Each condition was tested in triplicate. Mean + SD, **** p < 0.0001.

**Figure S8. Correlation matrices. (A)** Pearson r correlation of the infection inhibition capability of all the tested sera (1:20 dilution) and their post-entry inhibition (PEI) against XBB.1 and XBB.1.5 variants. ** p < 0.01, **** p < 0.0001. (**B)** Spearman r correlation computed between S1 shedding, NT and PEI results of a selected cohort of sera (#1, #2, #4, #5, #6, #11, #13, #17, #19, #22, #24, #26, #29). ** p < 0.01.

**
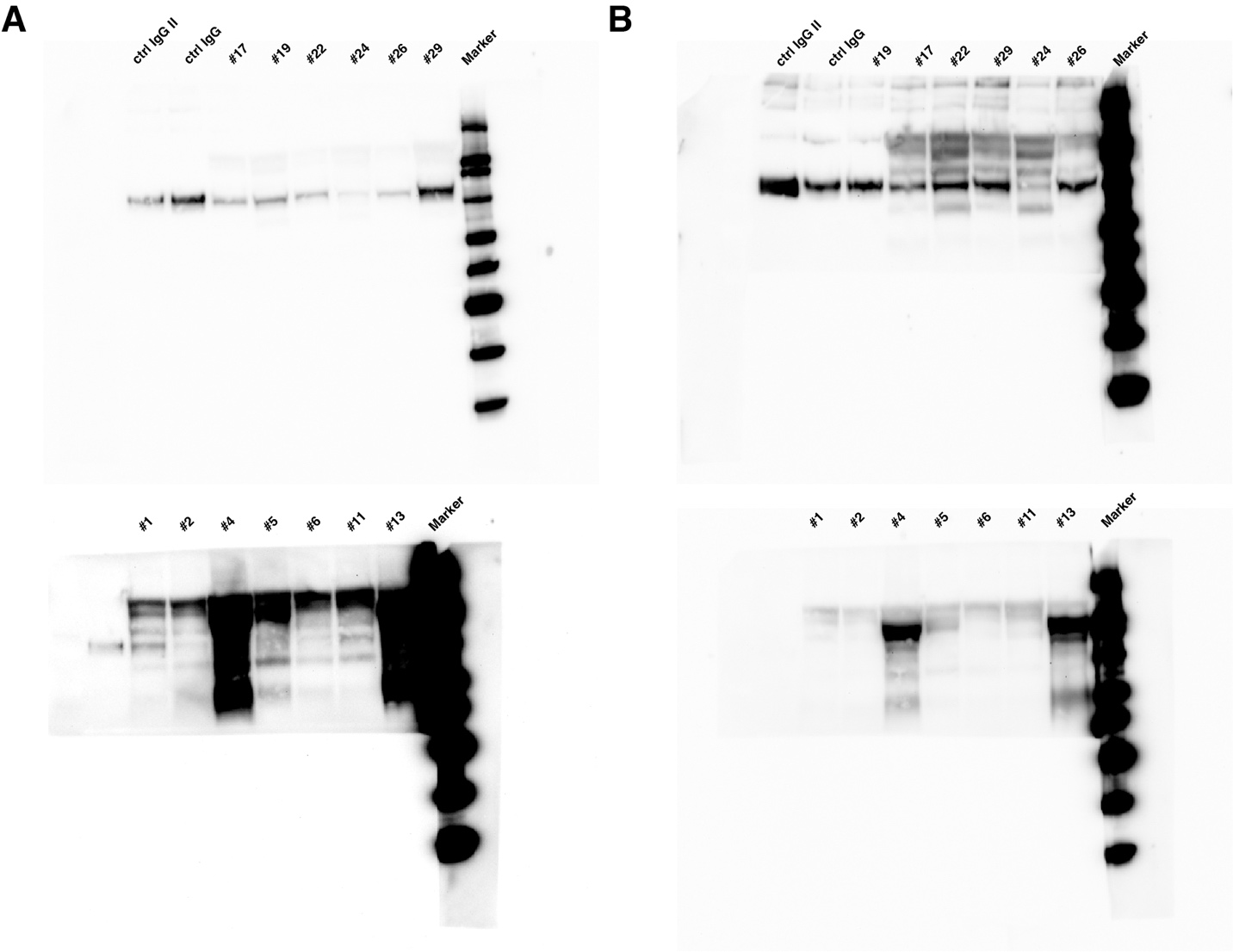
**

**Figure S9. Raw data for Western blots included in the Figure 5B.** Uncropped images of western blots on cells transfected with XBB.1 (**A**) or XBB.1.5 (**B**) recombinant S protein. The yellow boxes indicate the bands corresponding to the shedded S1 subunits.

**Table S1. Characteristics of serum donors whose samples were collected between July and October 2023.**

**Table S2. Sera neutralization of the XBB.1 variant.**

**Table S3. Sera neutralization of the XBB.1.5 variant.**
